# Quantitative analysis of piano performance proficiency focusing on difference between hands

**DOI:** 10.1371/journal.pone.0250299

**Published:** 2021-05-19

**Authors:** Sarah Kim, Jeong Mi Park, Seungyeon Rhyu, Juhan Nam, Kyogu Lee

**Affiliations:** 1 Music and Audio Research Group, Department of Intelligence and Information, Seoul National University, Seoul, South Korea; 2 Department of Transdisciplinary Studies, Seoul National University, Seoul, South Korea; 3 Graduate School of Culture Technology, Korea Advanced Institute of Science and Technology, Daejeon, South Korea; University of Pécs Medical School, HUNGARY

## Abstract

Quantitative evaluation of piano performance is of interests in many fields, including music education and computational performance rendering. Previous studies utilized features extracted from audio or musical instrument digital interface (MIDI) files but did not address the difference between hands (DBH), which might be an important aspect of high-quality performance. Therefore, we investigated DBH as an important factor determining performance proficiency. To this end, 34 experts and 34 amateurs were recruited to play two excerpts on a Yamaha Disklavier. Each performance was recorded in MIDI, and handcrafted features were extracted separately for the right hand (RH) and left hand (LH). These were conventional MIDI features representing temporal and dynamic attributes of each note and computed as absolute values (e. g., MIDI velocity) or ratios between performance and corresponding scores (e. g., ratio of duration or inter-onset interval (IOI)). These note-based features were rearranged into additional features representing DBH by simple subtraction between features of both hands. Statistical analyses showed that DBH was more significant in experts than in amateurs across features. Regarding temporal features, experts pressed keys longer and faster with the RH than did amateurs. Regarding dynamic features, RH exhibited both greater values and a smoother change along melodic intonations in experts that in amateurs. Further experiments using principal component analysis (PCA) and support vector machine (SVM) verified that hand-difference features can successfully differentiate experts from amateurs according to performance proficiency. Moreover, existing note-based raw feature values (Basic features) and DBH features were tested repeatedly via 10-fold cross-validation, suggesting that adding DBH features to Basic features improved F1 scores to 93.6% (by 3.5%) over Basic features. Our results suggest that differently controlling both hands simultaneously is an important skill for pianists; therefore, DBH features should be considered in the quantitative evaluation of piano performance.

## Introduction

The quantitative evaluation of a piano performance is very difficult due to the complex characteristics of music performances, in which the performer’s performance skills are expressed in alignment with his or her subjective interpretation of the music [1]. Moreover, except for the accuracy of pitch and rhythm, there is no explicit indicator that determines whether music is performed well [2, 3]. Such complex aspects of musical performance hinder objective evaluation; thus, human evaluation is generally involved. The human evaluation process is guided by standard grading scales and objective evaluation index rubrics [4].

In music education, evaluation is based on the opinion of highly trained professionals who produce subjective interpretations of the performance [5, 6]. However, even experienced evaluators can be biased since diverse musical experiences can lead to different interpretations of a piece of music, which could in turn result in a subjective evaluation [7, 8]. Despite these potential problems, subjective performance evaluations are common in music education [9]. Consequently, some studies have attempted to discover ways to quantitatively evaluate musical performances, and the implementation of an automatic performance evaluation system is considered to be an important task [10].

Most approaches to assessing musical performance use expertise to derive handcrafted features and then apply classification algorithms to predict expert ratings [9, 11]. Music information retrieval (MIR) methods have been utilized in an attempt to estimate pitch accuracy [12], timing accuracy, rhythmic accuracy, tone quality, and the handcrafted features of complex attributes [13–16]. In [17], the authors provided an objective performance assessment by recording the correlation coefficients of the onset time, velocity, and duration of each accuracy type with the judge’s evaluation score. In [18], the authors classified notes as accurate or inaccurate using a Bayesian classifier based on pitch deviations and note onset and offset time deviations extracted from students’ musical performances. The authors suggested that these evaluation indicators and evaluation methods could be used to interpret piano performances quantitatively and intuitively. In [19], standard audio features and a support vector machine (SVM) classifier were used to classify the sound quality of a trumpet performance as "good" or "bad". In [20], 138 feature sets were designed based on the pitch contour of the vocal and instrumental performances of students; an SVM was then trained based on the selected features. The authors found that the features that describe the variability of note length and the similarity between the musical score and the audio were the most influential.

Performance assessment studies have been conducted on various instruments in previous research, and the features have been analyzed according to the characteristics of each musical instrument. For example, stringed instruments were analyzed in terms of tone, timbre, and vibrato quality [10, 21, 22]; woodwind instruments and brass instruments were analyzed in terms of tone and pitch accuracy [14, 19]; percussion instruments were analyzed in terms of rhythm accuracy, onset time, and intensity information [23, 24]; and vocal music was analyzed with respect to pitch accuracy and vibrato [20, 25]. Furthermore, piano was recorded for onset, MIDI velocity, and duration of each musical note, and the average correlation coefficient between the system-estimated scores and expert scores was investigated, or the performed score was predicted [17, 26, 27]. The above piano evaluation studies extracted and analyzed the features of each of the two hands based on each note or onset of piano performance. However, there is a limitation in that the tendency to use a note-centered approach to analyze the player’s performance did not sufficiently reflect the characteristics of the actual piano performance; the instrument is played with both hands simultaneously. In most cases, note-oriented features were extracted from each of the two hands; to the best of our knowledge, few automatic performance evaluation studies have focused on the difference between the two hands (DBH) when using both hands at the same time [28].

Utilizing both hands fluently is considered to be fundamental for a pianist [29, 30]. Due to the nature of the piano keyboard, the balance of high and low tones reflects how well both hands are controlled. For example, in piano performance, most of the melody parts are played with the right hand (RH), and the accompaniment parts are usually played with the left hand (LH) [31]. In addition, to feature the melody prominently in a performance, the keys that play the melody must be struck relatively harder, while the accompaniment must be played at a relatively lower volume [32]. A highly skilled pianist plays the accentuated notes not only louder but also approximately 20–30 *ms* before the other notes [33]. This phenomenon is called *melody lead* [33–38]. Whether this phenomenon is part of a pianist’s intentional expressive strategy [37] or is caused by the timing characteristics of most piano action (*velocity artifact*) [38] is unclear. These characteristics are expected to vary depending on proficiency: a highly experienced pianist will understand the characteristics of high and low tones in the piano keyboard, control the balance of both hands, and perform with precise timing and intensity. To achieve sufficient hand independence, pianists focus on deliberate practice for more than 10 years or more than 10,000 hours [38–41]. In addition, professional pianists attempt to show an increase in the speed and accuracy of finger movements [42]. This focus not only promotes the robustness of the motor skills that enable individual finger movements [43] but also demonstrates piano skill and musical expression in their performances while making extremely elaborate movements with both hands.

Previous studies have shown the importance of two-handed performance feedback in acquiring musical skills [44]. Studies have explored ways to capture the physical position and movement of pianists for the analysis of piano performance and the assessment of automatic performance. They captured and analyzed the necessary performance data by utilizing motion capture systems, video recording or surface electronic graphics [44, 45]. Johnson *et al*. [44] performed segmentation of pianist hand posture to help beginning piano students to improve their piano playing skills during practice sessions and classified the correct hand posture by dividing the right and left hands using imaging features. Hadjakos *et al*. [46], using RGB video, presented three methods for hand assessment, detecting which hand played a note. Oka *et al*. [47] combined information from depth recording using Kinect and MIDI data to identify the pianist’s fingering mistakes. The aforementioned studies focused on the hands’ postures or movements and connected them to various experimental data including performance MIDI recordings. However, performance MIDI data have not been deeply explored as important media for examining hand differences when measuring performance proficiency.

Hence, the main objective of this study was to explore and analyze piano performance characteristics according to proficiency level in the context of quantitative performance evaluation. In particular, focusing on the two-hand relationship between experts and amateurs, the difference between the right and left hands was independently compared and analyzed using the entire performance. Specifically, we collected and analyzed performer data for two music pieces of similar styles that use two technical excerpts (Hanon Exercise No. 1 and a C-Major scale). The two hands play in unison in most of the exercises. We used algorithms to obtain information on the right and left hands during the performance and extracted features for both hands. In addition, further analysis with principal component analysis (PCA) and SVM were performed to obtain a variety of features to investigate this difference.

## Materials and methods

In this section, we explain the methods and procedures used for dataset acquisition and the features utilized in the statistical analyses and classification experiments. Then, the analysis methods are introduced.

### Dataset acquisition

#### Ethics statement

Every experimental protocol was approved by the Institutional Review Board (IRB) of Seoul National University. The experiments were performed in accordance with the relevant guidelines and regulations. Written consent forms were collected from the participants, and the study was conducted according to the ethical standards outlined in the 1962 Helsinki Declaration.

#### Participants

We recruited 34 experts (*mean* age = 23.75, *SD* = 2.96) and 34 amateurs (*mean* age = 24.67, *SD* = 3.65) for our experiment. The overall average age was 24.22 years old (*range* 20–37, *SD* = 3.33), and all of the participants were right-handed. The experts (28 women and 6 men) majored in piano performance at Seoul National University (32 individuals) or other universities (2 individuals). They started playing the piano at 6 years old on average (*SD* = 1.81) and had played for an average of 17.84 years (*SD* = 3.68) or 24,241 hours (*SD* = 8,194 hours) over their entire lives. The amateurs (14 women and 20 men) had a variety of majors and played piano as a hobby but had not studied the piano steadily. The amateurs had played the piano for an average of 12.48 years (*SD* = 4.52) but with an average of 3,970 hours (*SD* = 2,588) over their entire lives.

#### Materials

**Fig 1:**
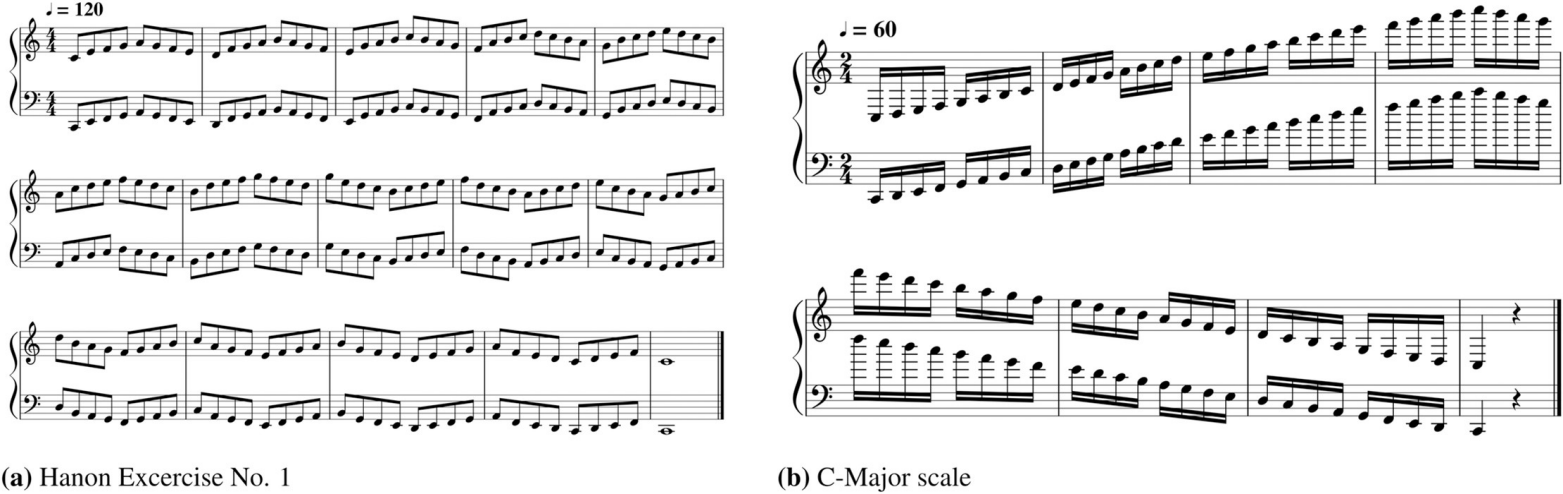
Musical excerpts: The music scores of the two excerpts selected for this study. (a) Hanon Exercise No. 1; (b) C-Major scale.

We utilized two piano excerpts (Hanon Exercise No. 1 and a C-Major scale) to be performed by the participants (Fig 1) because these piano excerpts can be played by both experts and amateurs, and because using other expressive musical pieces with specific styles can incur analytical bias or cause misinterpretation of the relationship between performance and musical proficiency [48, 49]. Each excerpt is simple but suitable for examining various evaluation categories, including rhythmic accuracy and articulation. In fact, the scale is a mandatory test for entrance examinations to music schools for piano majors in South Korea. Thus, the scale is a good musical excerpt to test a performer’s proficiency. A brief description of each excerpt follows.

- **Hanon Exercise No. 1:** This piece is a piano exercise for the two-handed building of technique. Charles-Louis Hanon (1819–1900) believed anyone who practices these exercises everyday could overcome any technical difficulties [50]. Starting from middle C (C4, middle of the piano keyboard, right hand) and the C two octaves below (C2, left hand), the performer plays a repeating eight-note pattern in ascending and descending sequence that spans one octave per hand. The excerpt requires the keys to be pressed with even velocity and duration and the use of a wide range of the keyboard, and it has patterns of repeated notes [51].
- **C-Major scale:** This excerpt is a basic element of musical structure not only in classical musical structure but also in several genres of jazz, rock and pop music [48, 49, 52]. It consists of a continuous C-Major scale played by both hands ascending and descending across four octaves. It is a combined motion, and it has a unique and fundamental difference from Hanon Exercise No. 1; playing the scale requires thumb crossover and cross-under control behaviors. As the fingering for scales differs between the right and left hands, this excerpt is particularly effective for observing the differences between the two hands with respect to temporal uniformity [53–55]. As a consequence, scale playing can represent a fundamental aspect of piano technique [52].

The total length of the data was 3,740 seconds, including, 2,040 seconds for Hanon Exercise No. 1 and 1,700 seconds for the C-Major scale. Since the Hanon Exercise No. 1 is written in eighth notes and the C-Major scale is written in sixteenth notes, we assigned 120 beats per minute (bpm) and 60 bpm for the excerpts, respectively, to analyze the two excerpts at the same tempo ratio (the same number of notes played per minute). Participants exhibited inter-subject tempo variation because the metronome was shown before the performance and not during the performance. Finally, the order in which the excerpts were played was randomly assigned to each pianist.

#### Procedure and apparatus

The participants played the 2 excerpts on a *Yamaha Disklavier C7X (DC7X ENPRO)* in a recording studio at the College of Music of Seoul National University. The *Yamaha Disklavier* is a concert-quality acoustic grand piano with a high-precision music instrument digital interface (MIDI) capture and playback system. While the participants were playing, MIDI data were recorded through the *Yamaha Disklavier* system. All of the performances were recorded in the same environment.

### Musical features

Objectively identify the characteristics of various piano performance techniques. In particular, performers’ piano skills can be assessed by measuring the tempo, timing, articulation, and dynamics [56]. Most previous studies have utilized the features of both hands together or each hand individually, but we additionally considered the differences between the two hands for each feature. We extracted features from the MIDI data recorded by the *Yamaha Disklavier*. In addition, we analyzed the features extracted from both hands separately to examine the balance between each performer’s hands. The balance was investigated using musical elements that are also used as indicators of piano playing technique, and the difference was calculated by extracting and comparing the features of the right and left hands. To this end, handcrafted features were extracted from the collected performance data in MIDI format. In particular, we defined those features based on those described by Bernays *et al*. [28], in which the factors of piano timbre nuance were investigated through extracting various performance features including hammer velocity or attack DBH.

#### Feature descriptions

We initially extracted conventional handcrafted features that represent the properties of piano performance. Table 1 describes each feature in detail. Most of these features are handcrafted features that denote the high-level properties of piano performance [57]. In this paper, the handcrafted features were computed to identify the explicit DBH. To this end, we initially extracted the conventional handcrafted features as the baselines, specifically, the Basic features, especially regarding the hands, as follows: both hands (BH), the right hand (RH), and the left hand (LH). RH and LH represent the value of each hand, and BH stands for the average of the right and left hands. Then, we utilized these Basic features with additional conventional features to compute the direct DBH, which resulted in another type of feature, namely, the DBH features. These two types of features were divided into temporal attributes and dynamic attributes. In this section, we describe the Basic and the DBH features in more detail.

**Table 1:** Description of the features used for analysis.

Features	Type	Name	Description
**Temporal**	BH	rDuration	The ratio of the duration of a performed note to the duration of the note written in the music score.
		rDuration Delta	The difference between a current note and previous note in rDuration.
	RH	rIOI	The ratio of the IOI of a performed note to the duration of the note written in the music score.
	LH		
		rIOI Delta	The difference between a current note and previous note in rIOI.
		Articulation	The ratio of the duration of a performed note to the IOI of a performed note.
		Articulation Delta	The difference between a current note and previous note in Articulation.
	DBH	Duration	The difference between hands of the performed note in duration.
		Duration Delta	The difference between a current note and previous note in DBH Duration.
		Attack Deviation	The difference between the hands that played the note.
		Attack Deviation Delta	The difference between a current note and previous note in Attack Deviation.
		Articulation	The difference between the hands of the ratio of the performed Duration note value and the performed note IOI value.
		Articulation Delta	The difference between a current note and previous note in Articulation value.
**Dynamics**	BH	Velocity	The loudness of a performed note.
	RH	Velocity Delta	The difference between a current note and previous note in Velocity value.
	LH		
	DBH	Velocity	The difference between hands of the performed key-attack velocity.
		Velocity Delta	The difference between a current note and previous note in DBH Velocity value.

The temporal-related features for BH, the RH, and the LH include the duration ratio (rDuration), rDuration Delta, inter onset interval ratio (rIOI), rIOI Delta, Articulation, and Articulation Delta, resulting in 18 (= 3 x 6), for the Basic features. For the DBH features, 6 features—Duration, Duration Delta, Attack Deviation, Attack Deviation Delta, Articulation, and Articulation Delta—are extracted. The dynamics-related features are indicators of the loudness of each note being played. They include the MIDI Velocity of a note and the Velocity Delta in BH, the RH, the LH and DBH, respectively, resulting in 16 (= 4 x 4) features. For all of the features, we compute the mean and standard deviation (SD) of each feature.

*Basic features*. These features include the temporal and dynamic attributes of musical elements. Each of the values in the handcrafted features was examined to analyze the timing and dynamics. We examined the rDuration, rIOI, Articulation, Duration, and the Delta values for these features to analyze the timing when attacking the keyboard with the RH and LH. For the measurement of dynamics, we utilized MIDI velocities.

- *rDuration*: This feature measures the ratio of the duration of a performed note to the duration of the note written in the musical score. This ratio was used to normalize the values across different note types (e.g., quarter notes versus eighth notes). The ratio was transformed into a logarithmic scale [58].
- *rIOI*: This class of features is the ratio of the interval between the beginning of consecutively played notes to the interval between the notes written in the score (i.e., the time between the beginning of one note and the beginning of the next). We covered polyphonic music, but to analyze the difference between the RH and LH, the IOI of a note was calculated as the distance between the current note and the next note for each hand. Specifically, the distance between the current note for the RH and the next note for the RH and the distance between the current note for the LH and the next note for the LH were calculated. The ratio was transformed into a logarithmic scale.
- *Articulation*: Articulation is a technique that affects the conversion or continuity between notes or sounds [59]. It is defined as the ratio of the performed note’s duration to the performed note’s IOI [60]. As above, these features were converted to a logarithmic scale.
- *Velocity*: We obtained the MIDI velocity values via the *Yamaha Disklavier*. Velocity measures the loudness of the performed note and is controlled by the pressure of the finger attacking the keyboard.

*Differences Between Hands (DBH) features*. Usually, a piano performer plays the melody and accompaniment parts with different hands. In general, the RH plays the melody, while the LH plays the accompaniment. If the balance between the two hands is adjusted well, the melody and accompaniment expressed by the performer are clearly divided and delivered to the audience. In this study, to examine the differences in the feature values between hands, the handcrafted features were regarded separately for the RH and LH. In contrast to most previous studies, in which the tempo and dynamics were analyzed by combining hands, we analyzed the RH and LH separately. We subtracted the left-hand values from the right-hand values to investigate this difference [61].

- *DBH Duration*: This feature is the difference between the duration of the right-hand note and the duration of the left-hand note during the performance.
- *DBH Attack Deviation*: This feature is the difference between the onset time of a note during the performance with the RH and the onset time of the note played by the LH.
- *DBH Articulation*: This feature is the ratio of the performance note duration to the performance note IOI, which is the difference between the right-hand and left-hand performance of simultaneous notes.
- *DBH Velocity*: This feature is the difference between the right-hand velocity and left-hand velocity of performed simultaneous notes based on MIDI velocity.

#### Feature extraction

To extract the feature differences between the two hands, MIDI sequences were aligned with score descriptors that indicated measures, information about musical symbols, and information about the RH and LH. We first converted the music scores into MusicXML format, including the Western music notation in its entirety. Then, we aligned the music scores with the recorded MIDI performance data using a state-of-the-art automatic alignment algorithm that connected the MusicXML and MIDI object sequences [62]. After alignment, we extracted all of the performance features (32), namely, in BH, the RH, and the LH, the rDuration, rDuration Delta, rIOI, rIOI Delta, Articulation, Articulation Delta, Velocity, and Velocity Delta (3 x 8 = 24) and in DBH, the Duration, Duration Delta, Attack Deviation, Attack Deviation Delta, Articulation, Articulation Delta, Velocity, and Velocity Delta (8) for each note. In addition, we extracted the measure numbers and staff numbers since they could be used to infer which note was played by which hand.

### Data analyses

#### Statistical analyses

We first performed statistical analyses to determine whether there is a significant difference between the proficiency groups for each factor. To assess the significance of the differences between the features for the two hands and for the two proficiency groups in the excerpts, the data were analyzed using mixed model analyses of variance (ANOVAs) with the features (rDuration, rIOI, Articulation, Velocity, rDuration Delta, rIOI Delta, Articulation Delta, and Velocity Delta) as the dependent variables, hands (left and right) as the within-subjects factor, and professionality (experts or amateurs) as the between-subjects factor. As a post hoc analysis, we conducted the Bonferroni correction for multiple comparisons. As a post hoc analysis to adjust for multiple comparisons and reduce false positives, we conducted a Bonferroni correction that yielded a corrected p-value of independent t-test. The p-values adjusted for readability were calculated by multiplying each value by the total number of multiple comparisons. An adjusted p-value less than .05 denotes significance [63]. In addition, Cohen’s *d* [64] coefficient was calculated to determine the magnitude of the effect between RH and LH in each group. For all DBH features (DBH Duration, DBH Attack Deviation, DBH Articulation, DBH Velocity, DBH Duration Delta, DBH Attack Deviation Delta, DBH Articulation Delta, and DBH Velocity Delta), we conducted independent t-tests between groups because the values represent the differences between hands. Levene’s equivariance test and normality tests were also performed. Statistical analyses were performed for each excerpt (Hanon and C-Major scale) and all excerpts.

### Classification

We further examined whether the extracted features that represent DBH could benefit classification tasks to distinguish between the proficiency groups. To this end, the following three types of data groups were organized depending on the composition of the different features: a group with 48 features including features for BH, the RH, and the LH (Basic); a group with 16 DBH features (DBH); and a group with all 64 features (Basic *+* DBH). The first two groups represented the baselines for our experiments, and the last group, which used all of the features, was our experimental group. Each set of data for the three groups was normalized by the corresponding Z scores, and PCA was conducted for each group. PCA is a standard feature analysis or feature reduction technique that finds new orthogonal axes that represent the maximum variance of the target data with the correlated variables. After finding the principal components, we sorted them in descending order by the amount of explained variance of the observed data so that the first component was the one that explains the largest ratio of the total variance. For classification, SVM with the linear kernel (*C* = 1.0) was used for a 10-fold cross-validation. SVM is a binary identification classifier that finds the optimal decision plane among the training feature vectors of different classes [65], and it has been widely used in studies of music performance evaluation. For a deeper analysis regarding the robustness of classification performance, we conducted the SVM training with a reduced number of principal components in three conditions. The first and second conditions have a certain number of principal components that cumulatively explain 95% and 99% of the total variance of the observed data, respectively, and the third condition is when the best classification score is shown regardless of the amount of cumulative explained variance. Furthermore, the number of features differs among the three feature sets; thus, the feature dimension itself can affect the classification scores among the feature sets. To disregard this factor, we conducted another experiment with the SVM in the cases of choosing various numbers of principal components that are constant across the feature groups [59], which are 10, 20, and 40. The overall process for PCA and SVM were implemented with the Scikit-Learn Library and LibSVM software, respectively [66, 67].

## Results

The results section comprises two parts, namely, the statistical analyses and classification result analyses. In the first part, the results show the factors that are significant to differentiate between the experts and amateurs using statistical analyses. In the second part, we compare the classification results among the 3 combinations of features (Basic + DBH, Basic, and DBH).

### Statistical analyses

**Fig 2:**
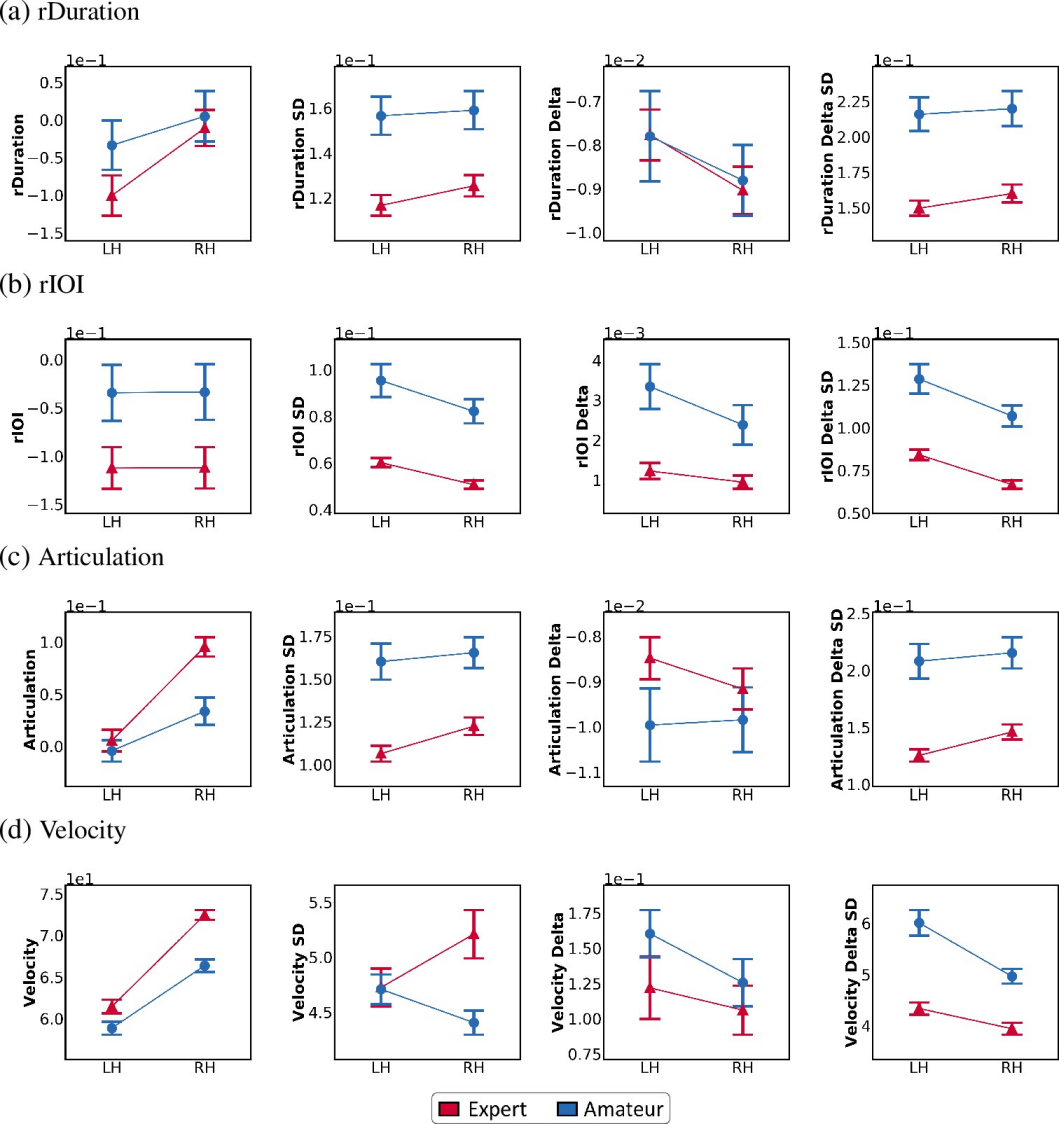
Comparison between hands for the Basic features in the expert and amateur groups. These line graphs show the main effects for both groups. The graphs are the results of an average of the feature values from both excerpts ("Hanon" and "Scale"). (a) The rDuration features as the mean, SD, Delta mean, and Delta SD. (b) The rIOI as the mean, SD, Delta mean, and Delta SD. (c) The Articulation features as the mean, SD, Delta mean, and Delta SD. (d) Velocity as the mean, SD, Delta mean, and Delta SD (expert, red; amateur, blue).

**Fig 3:**
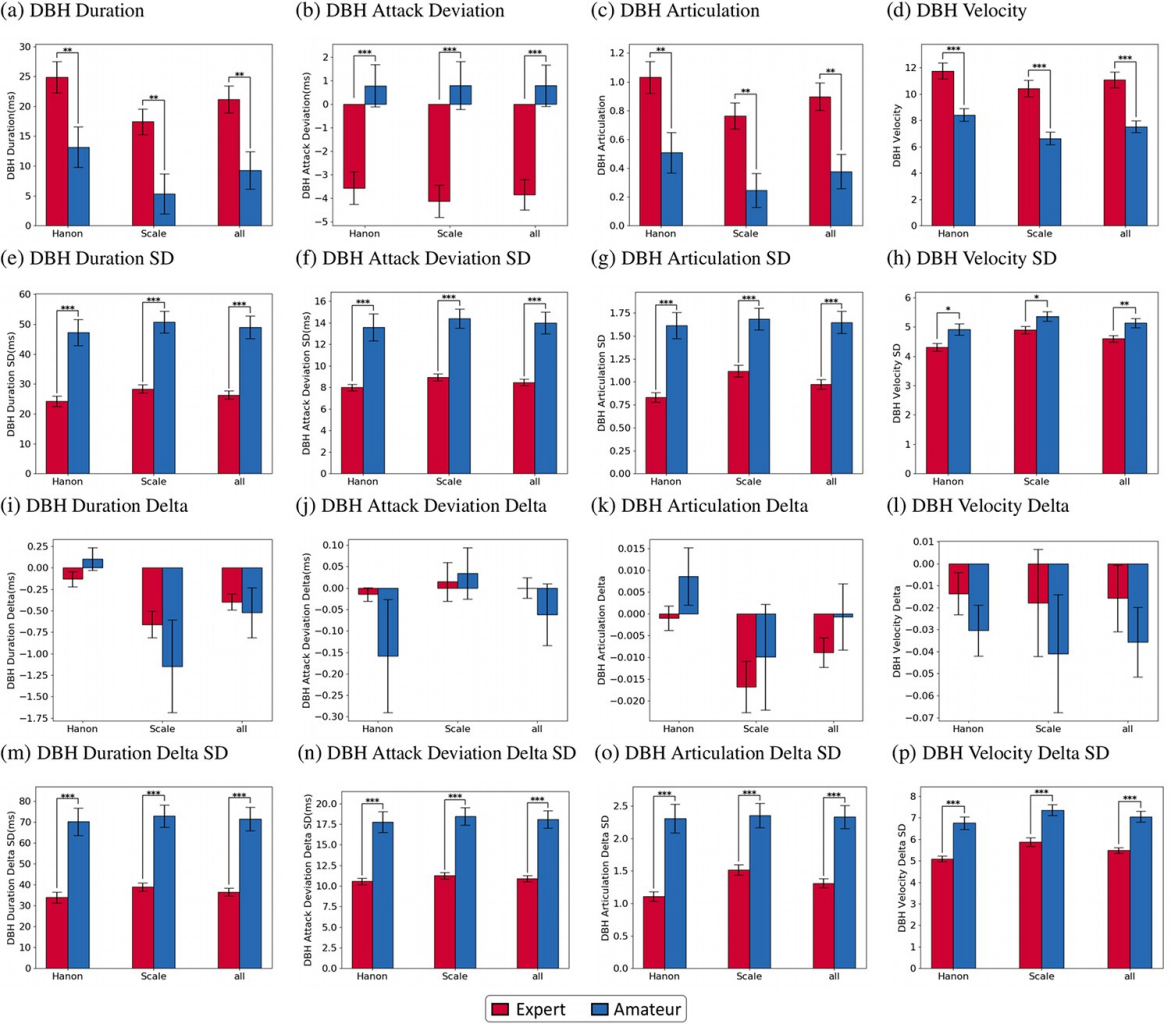
The bar plots with the results of the independent t-test for comparison of the DBH features. (a)-(d) The DBH features value of the mean; (e)-(h) the DBH features value of SD; (i)-(l) the DBH Delta features value of the mean; (m)-(p) the DBH Delta features value of SD. The DBH features were calculated as follows: right-hand notes—left-hand notes. A negative value indicates that the right hand tends to hit note(s) faster and for a shorter duration than the left hand when both hands play simultaneously, and a positive value indicates the opposite. Error bars represent standard errors (expert, red; amateur, blue). ***p < .001, **p < .01, *p < .05.

As mentioned in the previous section, rDuration, rIOI, Articulation, rDuration Delta, rIOI Delta, Articulation Delta, Velocity, and Velocity Delta were assessed via mixed model ANOVAs for each excerpt (Hanon, Scale, and all). The results are shown in Tables 2 and 4 and Fig 2. Additionally, the differences in the DBH features between hands were assessed via independent t-tests and were analyzed for each excerpt (Hanon, Scale, and all). The results are shown in Tables 3 and 5 and Fig 3.

**Table 2:** *F* -values and *p*-values in temporal features.

Features	Excerpts	Hand	*η*_*ρ*_^2^	Group	*η*_*ρ*_^2^	Hand x Group	*η*_*ρ*_^2^
rDuration	Hanon	76.684 (.000)***	.537	0.008 (.929)	.000	8.569 (.005)*	.115
	Scale	46.289 (.000)***	.412	2.895 (.094)	.042	11.591 (.001)**	.149
	all	70.453 (.000)***	.516	1.02 (.316)	.015	11.305 (.001)**	.146
rDuration SD	Hanon	0.434 (.512)	.007	21.304 (.000)***	.244	0.765 (.385)	.011
	Scale	9.628 (.003)**	.127	4.784 (.032)*	.068	0.412 (.523)	.006
	all	2.678 (.107)	.039	16.581 (.000)***	.201	0.842 (.362)	.013
rIOI	Hanon	1.562 (.216)	.023	0.768 (.384)	.012	0.008 (.928)	.000
	Scale	0.8 (.374)	.012	8.901 (.004)**	.119	0.420 (.519)	.006
	all	1.91 (.172)	.028	4.72 (.033)*	.067	0.309 (.58)	.005
rIOI SD	Hanon	18.492 (.000)***	.219	9.746 (.003)**	.129	1.273 (.263)	.019
	Scale	7.935 (.006)**	.107	27.976 (.000)***	.298	0.000 (1)	.000
	all	24.471 (.000)***	.27	30.694 (.000)***	.317	0.675 (.414)	.01
Articulation	Hanon	75.26 (.000)***	.533	2.371 (.128)	.035	8.645 (.005)**	.116
	Scale	47.269 (.000)***	.417	14.462 (.000)***	.18	12.293 (.001)**	.157
	all	70.71(.000)***	.517	7.177 (.009)**	.098	11.709 (.001)**	.151
Articulation SD	Hanon	0.93 (.761)	.001	21.844 (.000)***	.249	3.378 (.071)	.049
	Scale	12.909 (.001)**	.164	13.399 (.001)**	.169	0.003 (.959)	0
	all	5.287 (.025)*	.074	23.592 (.000)***	.263	1.355 (.249)	.02
rDuration Delta	Hanon	1.027 (.314)	.015	4.966 (.029)*	.07	2.31 (.133)	.034
	Scale	9.219 (.003)**	.123	2.574 (.113)	.038	0.018 (.893)	.000
	all	8.984 (.004)**	.12	0.001 (.992)	.000	0.121 (.729)	.002
rDuration Delta SD	Hanon	0.345 (.559)	.005	26.153 (.000)***	.284	0.964 (.33)	.014
	Scale	6.587 (.013)*	.091	11.856 (.001)**	.152	0.003 (.955)	0
	all	1.843 (.179)	.027	26.28 (.000)***	.285	0.371 (.544)	.006
rIOI Delta	Hanon	16.086 (.000)***	.196	2.24 (.139)*	.033	5.567 (.021)*	.078
	Scale	7.973 (.006)**	.108	13.359 (.001)**	.168	2.239 (.139)	.033
	all	13.059 (.001)**	.165	11.162 (.001)**	.145	3.934 (.051)	.056
rIOI Delta SD	Hanon	22.894 (.000)***	.258	12.446 (.001)**	.159	0.871 (.354)	.013
	Scale	10.137 (.002)**	.133	32.379 (.000)***	.329	0.021 (.887)	0
	all	30.922 (.000)***	.319	34.433 (.000)***	.343	0.359 (.551)	.005
Articulation Delta	Hanon	0.592 (.444)	.009	1.118 (.294)	.017	1.466 (.23)	.022
	Scale	0.846 (.361)	.013	1.345 (.025)	.02	0.424 (.517)	.006
	all	0.384 (.537)	.006	2.001 (.162)	.029	0.767 (.384)	.011
Articulation Delta SD	Hanon	0.024 (.878)	.000	23.391 (.000)***	.262	3.730 (.058)	.053
	Scale	11.526 (.001)**	.149	20.498 (.000)***	.237	0.230 (.633)	.033
	all	4.310 (.042)*	.061	29.172 (.000)***	.307	0.953 (.333)	.014

“all” represents an average of the feature values from the two excerpts ("Hanon” and “Scale”).

***p < .001

**p < .01

*p < .05.

**Table 3:** Independent t-test statistics and *p*-value of the two groups (expert and amateur).

Features	Excerpts	Groups				t-values (*p*)
		Expert		Amateur		
		mean	SD	mean	SD	
DBH Duration	Hanon	0.025	0.015	0.013	0.0198	-2.724 (.008)**
	Scale	0.017	0.013	0.005	0.0197	-3.003 (.004)**
	all	0.021	0.013	0.009	0.018	-3.074 (.003)**
DBH Duration SD	Hanon	0.024	0.0103	0.047	0.025	4.898 (.000)***
	Scale	0.028	0.008	0.051	0.021	5.815 (.000)***
	all	0.026	0.008	0.049	0.023	5.590 (.000)***
DBH Attack Deviation	Hanon	-0.004	0.004	0.008	0.005	3.835 (.000)***
	Scale	-0.004	0.004	7.954e-4	0.006	4.004 (.000)***
	all	-0.004	0.004	7.906-e4	0.005	4.251 (.000)***
DBH Attack Deviation SD	Hanon	0.008	0.002	0.014	0.007	4.364 (.000)***
	Scale	0.009	0.002	0.014	0.005	5.810 (.000)***
	all	0.008	0.002	0.014	0.006	5.241 (.000)***
DBH Articulation	Hanon	0.103	0.064	0.051	0.082	-2.942 (.005)**
	Scale	0.076	0.053	0.025	0.069	-3.452 (.001)**
	all	0.090	0.056	0.038	0.070	-3.387 (.001)**
DBH Articulation SD	Hanon	0.083	0.031	0.161	0.083	5.168 (.000)***
	Scale	0.112	0.037	0.168	0.069	4.195 (.000)***
	all	0.097	0.031	0.165	0.071	5.102 (.000)***
DBH Duration Delta	Hanon	-1.324-e4	5.225-e4	1.026-e4	7.565-e4	1.490 (.141)
	Scale	-6.605-e4	9.056-e4	-0.001	0.003	-0.869 (.388)
	all	-3.964-e4	5.441-e4	-5.227-e4	0.002	-0.413 (.681)
DBH Duration Delta SD	Hanon	0.034	0.015	0.071	0.038	5.136 (.000)***
	Scale	0.039	0.011	0.073	0.031	6.054 (.000)***
	all	0.036	0.011	0.072	0.033	5.894 (.000)***
DBH Attack Deviation Delta	Hanon	-1.484-e5	9.183-e5	-1.586-e4	7.714-e4	-1.079 (.285)
	Scale	1.471-e5	2.622-e4	3.376-e5	3.490e-4	0.255 (.800)
	all	-6.565-e8	1.385-e4	-6.240-e5	4.201-e4	-0.822 (.414)
DBH Attack Deviation Delta SD	Hanon	0.011	0.002	0.018	0.007	5.409 (.000)***
	Scale	0.011	0.002	0.018	0.006	6.373 (.000)***
	all	0.012	0.002	0.018	0.006	6.395 (.000)***
DBH Articulation Delta	Hanon	-1.004-e4	0.002	8.557-e4	0.004	1.341 (.185)
	Scale	-0.002	0.003	-9.904-e4	0.008	0.512 (.610)
	all	-8.908-e4	0.002	-6.735-e5	0.004	0.991 (.325)
DBH Articulation Delta SD	Hanon	0.111	0.044	0.231	0.129	5.135 (.000)***
	Scale	0.152	0.048	0.235	0.108	4.129 (.000)***
	all	0.131	0.04	0.233	0.103	5.373 (.000)***

The features labeled “all” represent an average of the feature values from the two excerpts ("Hanon” and “Scale”).

***p < .001

**p < .01

*p < .05.

#### Temporal attributes

We examine the temporal attributes in the Basic features, including rDuration, rIOI, and Articulation, of the performance from the two proficiency groups. First, Table 2 and Fig 2 show that the temporal attributes indicated a significant main effect of hand in all types of excerpts (*p* < .05), except for the rIOI mean. In addition, the main effect of group is shown to be significant in all excerpts for rDuration SD [all excerpts: *F* (1, 66) = 16.581, *p* < .001, *η*_*ρ*_^2^ = .201], rIOI SD [all excerpts: *F* (1, 66) = 30.694, *p* < .001, *η*_*ρ*_^2^ = .317] and Articulation SD [all excerpts: *F* (1, 66) = 23.592, *p* < .001, *η*_*ρ*_^2^ = .263]. The interaction effect between hand and group is significant only for the rDuration mean and Articulation mean (*p* < .05). After applying post hoc tests with Bonferroni’s correction to comparisons between hands in each group (expert or amateur group), we found significant differences in both groups, but the results were more significant with a fairly large effect size in the expert group (*p* < .001; Cohen’s *d* = .606 for rDuration mean and *p* < .001; Cohen’s *d* = 1.564 Articulation mean) than in the amateur group (*p* = .017; Cohen’s *d* = .2 for rDuration mean, *p* = .019; Cohen’s *d* = .551 for Articulation mean).

The Delta features of the temporal attributes showed different results. The main effect of hand is significant in all excerpts for the rIOI Delta mean [all excerpts: *F* (1, 66) = 13.059, *p* < .001, *η*_*ρ*_^2^ = .165] and rIOI Delta SD [all excerpts: *F* (1, 66) = 30.922, *p* < .001, *η*_*ρ*_^2^ = .319]. All attributes showed a significant main effect for the group in all excerpts (*p* < .05), except for the rDuration Delta mean and Articulation Delta mean. The interaction effect was significant only for the rIOI Delta mean in the Hanon excerpt [*F* (1, 66) = 5.667, *p* < 0.05, *η*_*ρ*_^2^ = .078]. After applying Bonferroni’s correction post hoc tests to each group, we found that DBH was significant only in the amateur group (*p* = .007) but not in the expert group (*p* = .462). Accordingly, the main effect of hand is explicit in all types of excerpts for the rIOI SD, indicating that the variance of the rIOI differs significantly between the two hands regardless of group. Furthermore, the main effect of group appears to be clear in all types of excerpts for the rDuration SD, rIOI mean and SD, Articulation SD, and rIOI Delta mean and SD. This outcome indicates that the two proficiency groups show significantly different values for these features regardless of hand, while the significant interaction between the rDuration mean and Articulation mean demonstrates that the main effects of hand and group significantly affect one another.

For the DBH features, the non-Delta temporal attributes mostly showed a significant difference between the two proficiency groups (*p* < .01), as revealed in Table 3. First, the DBH Duration features were significantly different between the two groups [DBH mean (all excerpts): *t* (66) = -3.074, *p* < .001; DBH SD (all excerpts): *t* (66) = 5.590, *p* < .001]. The DBH Attack Deviation features showed a similar trend in that the differences between proficiency groups were significant [DBH mean (all excerpts): *t* (66) = 4.251, *p* < .001; DBH SD (all excerpts): *t* (66) = 5.241, *p* < .001]. The DBH Articulation features also significantly differed between the experts and amateurs [DBH mean (all excerpts): *t* (66) = -3.387, *p* < .001; DBH SD (all excerpts): *t* (66) = 5.102, *p* < .001]. The DBH Articulation features also significantly differed between the experts and amateurs [DBH mean (all excerpts): *t* (66) = -3.387, *p* < .001; DBH SD (all excerpts): *t* (66) = 5.102, *p* < .001]. These results show that the temporal attributes regarding DBH in actual duration, actual attack time, and how well the duration follows the score in personal tempo are significantly different between the expert and amateur groups in both their means and SDs. For the Delta features, in contrast, all of the DBH Delta mean features of the temporal attributes were not significant in distinguishing experts from amateurs, compared to the DBH Delta SD features. The DBH Duration Delta SD showed a significant difference between the two groups [DBH Delta SD (all excerpts): *t* (66) = 5.894, *p* < .001], whereas the corresponding Delta mean did not show a significant difference [DBH Delta Mean (all excerpts): *t* (66) = -0.413, *p* = .681]. The proficiency groups also significantly differed in the DBH Attack Deviation Delta SD [DBH Delta SD (all excerpts): *t* (66) = 5.894, *p* < .001] but not in the corresponding Delta mean [DBH Delta mean (all excerpts): *t* (66) = -0.822, *p* = .414]. Finally, the DBH Articulation Delta SD showed a significant difference between the two groups [DBH Delta SD (all excerpts): *t* (66) = 5.373, *p* < .001], but the corresponding Delta mean did not show a significant difference [DBH Delta mean (all excerpts): *t* (66) = 0.991, *p* = .325]. These results show that the variance of sequential changes in temporal properties differs significantly between the proficiency groups, whereas how they change on average does not differ significantly for the two proficiency groups.

#### Dynamic attributes

As shown in Table 4 and Fig 2, dynamic attributes in the Basic features tend to show a significant main effect of both hand and group, while they also have significant interaction effects between hand and group. In particular, the Velocity mean shows a significant main effect of hand and group for all types of excerpts [all excerpts (Hand): *F* (1, 66) = 614.359, *p* < .001, *η*_*ρ*_^2^ = .903; all excerpts (Group): *F* (1, 66) = 19.713, *p* < .001, *η*_*ρ*_^2^ = .23], as well as a significant interaction effect for all excerpts [all excerpts: *F* (1, 66) = 21.795, *p* < .001, *η*_*ρ*_^2^ = .248]. After applying Bonferroni’s post hoc correction, we found that the DBH in each group was significant (Bonferroni, *p <* .001; Cohen’s *d* = 2.624 for experts, *p <* .001; Cohen’s *d* = 1.697 for amateurs). The Velocity SD shows a less significant main effect of hand and group than the Velocity mean, indicating this effect only for subsets of the excerpts. There is also a significant interaction for the Velocity SD in all types of excerpts [all excerpts: *F* (1, 66) = 19.688, *p* < .001, *η*_*ρ*_^2^ = .23]. Post hoc tests using Bonferroni’s correction revealed that, while the expert group with RH and LH showed significant differences (*p* < .001), the amateur group did not.

**Table 4:** F-statistics and *p-*values for dynamic features.

Features	Excerpts	Hand	*η*_*ρ*_^2^	Group	*η*_*ρ*_^2^	Hand x Group	*η*_*ρ*_^2^
Velocity	Hanon	670.106 (.000)***	.91	13.133 (.001)**	.166	18.415 (.000)***	.218
	Scale	435.439 (.000)***	.868	23.196 (.000)***	.26	20.176 (.000)***	.234
	all	614.359 (.000)***	.903	19.713 (.000)***	.23	21.795 (.000)***	.248
Velocity SD	Hanon	6.669 (.012)*	.092	0.397 (.531)	.006	9.183 (.003)**	.122
	Scale	21.856 (.000)***	.249	9.943 (.002)**	.131	12.917 (.001)**	.164
	all	1.405 (.31)	.016	3.677 (.006)**	.053	19.688 (.000)***	.23
Velocity Delta	Hanon	8.708 (.004)**	.117	2.819 (.098)	.041	1.251 (.267)	.019
	Scale	2.662 (.108)	.039	0.626 (.432)	.009	0.374 (.543)	.006
	all	5.752 (.019)*	.08	1.482 (.228)	.022	0.816 (.37)	.012
Velocity Delta SD	Hanon	23.967 (.000)***	.266	45.421 (.000)***	.408	3.574 (.063)	.051
	Scale	13.307 (.001)**	.168	27.47 (.000)***	.294	3.908 (.052)	.056
	all	30.518 (.000)***	.316	47.247 (.000)***	.417	6.216 (.015)*	.086

“all” represents an average of the feature values from the two excerpts ("Hanon” and “Scale”).

***p < .001

**p < .01

*p < .05.

In contrast, the Delta features of the dynamic attributes appear to have different aspects from the non-Delta features. First, the Velocity Delta mean shows a significant main effect of hand only for the Hanon and all excerpts [Hanon: *F* (1, 66) = 8.708, *p* < .01, *η*_*ρ*_^2^ = .117, all excerpts: *F* (1, 66) = 5.752, *p* < .05, *η*_*ρ*_^2^ = .08]. There is no significant main effect of group in the Velocity Delta mean (*p* < .05) and no significant interaction effects (*p* < .1) between hand and group for all types of excerpts. The Velocity Delta SD, in contrast, shows a significant main effect of both hand and group for all types of excerpts [all excerpts (Hand): *F* (1, 66) = 30.518, *p* < .001, *η*_*ρ*_^2^ = .316; all excerpts (Group): *F* (1, 66) = 47.247, *p* < .001, *η*_*ρ*_^2^ = .417], with significant interactions only for all excerpts [*F* (1, 66) = 6.216, *p* < .05, *η*_*ρ*_^2^ = .086]. Post hoc tests using Bonferroni’s correction revealed that, the DBH was more significant, with a larger effect size in the amateur group (*p* < .001; Cohen’s *d* = -.875) than in the expert group (*p* = .031; Cohen’s *d* = -.584). Accordingly, the Velocity mean and SD features indicate the main effects of hand and group that can significantly influence one another, despite the effects’ significance. In contrast, the Velocity Delta SD establishes concrete, significant main effects of hand and group for the Hanon and Scale excerpts. The Velocity Delta SD for all excerpts has significant main effects of hand and group that are not significantly independent of each other.

The DBH features in the dynamic attributes, as shown in Table 5 and Fig 3, demonstrate a significant difference between the experts and amateurs, except for the DBH Velocity Delta mean. In particular, the two proficiency groups signal a significant difference in both the DBH Velocity mean and DBH Velocity SD [DBH mean (all excerpts): *t* (66) = -4.785, *p* < .001; DBH SD (all excerpts): *t* (66) = 2.802, *p* < .001]. This outcome indicates that the DBH in Velocity is significantly different between the two proficiency groups in both mean and variance. For the DBH Delta features, the DBH Velocity Delta SD shows similar aspects in that the experts and amateurs significantly differ in this feature [DBH Delta SD (all excerpts): *t* (66) = 5.641, *p* < .001], while DBH Velocity Delta mean does not significantly differ [DBH Delta mean (all excerpts): *t* (66) = -0.912, *p* = .365]. This finding indicates that the variance in the change in the DBH Velocity is significant in distinguishing the experts from amateurs, although the mean of the change is not significant.

**Table 5:** Independent-t test statistics and *p*-value of the two groups (expert and amateur).

Features	Excerpts	Groups				t-values (*p*)
		Expert		Amateur		
		mean	SD	mean	SD	
DBH Velocity	Hanon	11.754	3.571	8.411	2.807	-4.292(.000)***
	Scale	10.412	3.718	6.631	2.864	-4.699(.000)***
	all	11.083	3.472	7.521	2.606	-4.785(.000)***
DBH Velocity SD	Hanon	11.754	0.756	8.411	1.142	2.589(.012)*
	Scale	4.893	0.757	5.354	0.953	2.207(.031)*
	all	4.601	0.640	5.135	0.910	2.802(.007)**
DBH Velocity Delta	Hanon	-0.014	0.056	-0.030	0.068	-1.111(.271)
	Scale	-0.018	0.142	-0.041	0.157	-0.635(.527)
	all	-0.016	0.088	-0.036	0.092	-0.912(.365)
DBH Velocity Delta SD	Hanon	5.085	0.763	6.756	1.750	5.106(.000)***
	Scale	5.879	1.178	7.354	1.494	4.521(.000)***
	all	5.482	0.769	7.055	1.433	5.641(.000)***

Features labeled “all” represent an average of the feature values from the two excerpts ("Hanon” and “Scale”).

***p < .001

**p < .01

*p < .05.

As shown in Fig 3, the DBH Velocity value, which represents DBH, differed greatly between the groups [*t* (66) = -4.785, *p* < .001]. In all of the excerpts, both groups played the RH with a greater Velocity than the LH, but the experts showed a larger difference.

### Classification

In this section, we present the experimental results from the PCA and SVM for each of the three groups, namely the basic group with 48 Basic features in BH, RH, and LH; the DBH group with 16 DBH features; and the Basic + DBH group with all 64 features. The linear kernel SVM was trained via a 10-fold cross-validation with the extracted PCA components for each of the three groups. The classification results for the three different conditions are shown in Table 6. The results generally show that the mean *F* 1 scores using all features (Basic + DBH group) are higher than the other feature sets (Basic group and DBH group) in all three conditions (0.8687 for 95% explained variance; 0.9137 for 99% explained variance; 0.9361 for best score). After further experiments in which the number of principal components was fixed across the feature sets, the Basic + DBH group still revealed a greater mean *F* 1 score than the other feature sets, regardless of the number of the components, as shown in Table 7 (0.8760 for 10 components; 0.9281 for 20 components; 0.9135 for 40 components). Since the total number of the DBH features is 16, the DBH-only group is discarded in experiments with 20 and 40 components.

**Table 6:** The results of the linear SVM of the three groups of feature types (Base + DBH, Base, and DBH).

Condition	95% Explained Variance			99% Explained Variance			Best Classification Score		
Feature Set	Basic + DBH	Basic	DBH	Basic + DBH	Basic	DBH	Basic + DBH	Basic	DBH
# of PCA Components	17	13	10	26	20	13	20	9	13
Mean *F* 1	**0.8687**	0.8334	0.8502	**0.9137**	0.8813	0.8748	**0.9361**	0.9014	0.8748
Std *F* 1	0.0794	0.1044	0.0666	0.0679	0.0911	0.1192	0.0626	0.0746	0.1192

The table shows the number of PCA components used for classification, the mean *F* 1 score, and its standard deviation. The number of the principal components is chosen by the following cases: when the sorted components can explain 95% or 99% of the total variance of the observed data or can show the highest mean *F* 1 score regardless of the explained variance.

**Table 7:** The results of further experiments with the linear SVM on the three groups of feature types (Base + DBH, Base, and DBH).

# of PCA Components	10			20			40		
Feature set	Basic + DBH	Basic	DBH	Basic + DBH	Basic	DBH	Basic + DBH	Basic	DBH
Mean *F* 1	**0.8760**	0.8642	0.8568	**0.9281**	0.8813	-	**0.9135**	0.8785	-
Std *F* 1	0.1011	0.0895	0.1087	0.0774	0.0911	-	0.0785	0.0843	-

The table shows the mean *F* 1 score and its standard deviation from SVM, depending on the number of principal components (10, 20, and 40) fixed across the groups.

## Discussion

The present study examined whether it is necessary to regard the two hands separately for quantitative and objective assessment of piano performance. We investigated whether DBH is a statistically significant factor in distinguishing the expert group from the amateur group and examined its effect on accurate proficiency classification. To this end, we recorded piano performance participants according to their proficiency. Two excerpts, which were selected in consideration of the amateurs’ skill level, were obtained in MIDI format. We aligned the performance data (MIDI format) with the music scores (MusicXML format) and extracted handcrafted features that have previously been used for the quantitative assessment of piano performance. These features include the basic notewise features related to the temporal and dynamic attributes in the performance data and the features of the DBH that also correspond to performance attributes. We not only considered the explicit features for the hand difference but also subtracted the features for the two hands from one another so that the two hands could be explicitly regarded as separate factors. Through a statistical analysis, we identified DBH in the experts and amateurs in terms of temporal and dynamic attributes. In addition, a classification task with PCA and SVM showed the reliability of the features for DBH in a machine learning-based assessment of performance proficiency. Furthermore, the results obtained from this experiment support several studies observing the movement of the hand or body in playing the instrument, and they strongly demonstrate that performance behavior, performance characteristics, and proficiency are closely related [41, 43].

### Temporal attributes

From our analysis of the temporal attributes regarding the note duration, IOI, and articulation, we found several insights that led us to discover the importance of DBH when comparing the two proficiency groups of experts and amateurs. We computed the conventional handcrafted features from each note of the collected MIDI piano performances, which are denoted as the Basic features, and further derived the DBH features from the conventional features to investigate the direct differences between the two hands and how these differences appear in piano performances from the two proficiency groups.

First, we found that the Basic features could provide clues that experts and amateurs have different tendencies in how their two hands hit the notes together. In particular, it was found that the interaction effect between hand and group is significant for both the rDuration mean and Articulation mean in all types of excerpts, although the main effects of hand for the rDuration mean and the Articulation mean are also significant for all excerpts. Fig 2 shows that the horizontal lines between the LH and RH mean values are different in slope between experts and amateurs for both the rDuration mean and Articulation mean. Experts show a steeper slope between the RH and LH than amateurs for both the rDuration and Articulation. Intuitively, this outcome suggests that the experts hit the RH notes for a much longer duration than the LH notes compared to the amateurs. This trend is also evident in that both the DBH Duration mean and DBH Articulation mean are shown to be significantly greater for the experts than for the amateurs, as illustrated in Fig 3. The excerpts used in this study are in the form of unison in which both hands play the same melody and same note at the same time, but it is noted that the results of both hands are distinctly different in the expert group than in the amateur group. Although two notes of the same pitch and same length parallel on the one or two-octave difference are played simultaneously by the RH and LH, the results showing that experts play the RH note longer than the LH note supports previous studies’ argument that professional pianists tend to press the keys with their RH for a longer duration to emphasize melody [33, 68, 69]. In general, pianists do not strictly play both hands equally at the same time, despite the simultaneous notes indicated on the music score [33]. In addition, certain notes are played louder or longer to emphasize important events between consecutive pitches, simultaneous pitches, or harmonies [33, 37, 70]. Professional pianists tend to play longer with the RH than their LH in note onset asynchronous type (when the melody is on the right hand but not in the bass (typically left hand)) [71, 72]. In other words, this behavior can be regarded as an intentional expression device controlled directly by the pianist in the between-hands asynchronous playing. The results of this study suggest that the length of performance between both hands varies depending on proficiency, supporting previous studies of piano performance analysis showing that professional pianists tend to press the note with the RH for a longer duration than student pianists in order to emphasize the melody [37, 73]. Artistic music performances require motor skills for the performer to manipulate the musical elements [74]. According to Goebl *et al*. [41], piano performance essentially requires goal-oriented body movements. To produce the intended sound as precisely and accurately as possible using expressive parameters such as timing, dynamics, timbre, and articulation, pianists try to simultaneously stabilize motion kinematics in their hands and fingers [41]. Additionally, the effect of tempo adjustment on the movement configuration differed between experienced and inexperienced piano players and provided superior physiological efficiency and independent finger movement control to more experienced pianists [43]. We believe this difference to have resulted from the highly trained motor difference in the ability to control the length of the note, and our results show that investigating DBH for rDuration and Articulation can empirically help to distinguish experts from amateurs in performing simple exercises.

Moreover, the results for these two features differed in that the experts showed lower mean values for rDuration but higher mean values for Articulation than the amateurs (Fig 2). This result suggests that rDuration and Articulation can also provide different insights from one another, although they both implicitly represent how long the note is performed compared to the corresponding standard. These features differ with respect to their denominators. Articulation’s denominator is the performed IOI, which can vary among performers, hands, or the excerpt. On the other hand, the denominator for computing the rDuration is a quantized, absolute length, which comes from the original score itself. Therefore, rDuration and Articulation can be interpreted as the absolute tempo of a given score and the relative tempo of the performers, respectively. For instance, if someone plays a piece at a much faster tempo than the given tempo, the rDuration mean values can be small, although the player may believe that he or she has played the notes for the full length. Regarding these characteristics, a lower mean value of rDuration in experts indicates that experts tend to play the piece for a shorter note duration compared to the original quantized score. If the experts’ rIOI mean values are much lower than that of the amateurs, the results for the rDuration mean suggest that the experts might have played the notes at a faster tempo than the amateurs, resulting in a shorter note duration of the performed notes. However, higher values of Articulation from the experts’ piano performances indicate that the experts are better able than the amateurs to follow the modified score duration according to their personal tempo. That is, the experts might have played according to their personal tempo, while the amateurs might have played notes for a shorter duration than the length that they should have played them. Further, it could mean that the experts played the notes at a more suitable rate—even at a faster tempo—than amateurs. This outcome, similar to the previous interpretation, supports the previous finding that the finger movements of experts and amateurs differ when a given music piece is played at a fast tempo [43, 74]. Fundamentally, the compositions of keystroke hand movements of highly experienced pianists and novices are different [41]. Many previous studies have provided evidence for the basic kinematics of skilled musical performance [41, 42, 74–77]. According to Furuya *et al*. [43], professional pianists showed less increased mobility tissue muscles in the fingers and more in the elbow than amateur pianists, as well as increased endurance against fatigue by effectively using a larger proximal muscle. In addition, the effect of tempo adjustment on movement constitution differed between skilled piano performance and nonskilled piano performance and provided superior physiological efficiency and independent finger movement control for more skilled pianists [43]. This superb physical movement of a professional pianist allows the professional to reduce the loss of keystroke length associated with accurately pressing the piano keys at a fast tempo [74]. The above experimental results show a tendency of differences in Duration and Articulation between experts and amateurs to be distinguished, suggesting that experts are more proficient than amateurs in the physical exercise of playing the piano.

The SD features for DBH Duration and DBH Articulation are also shown to be significantly different between the two proficiency groups. In particular, the experts revealed significantly lower SD for these features than the amateurs. This finding suggests that DBH in the Duration and Articulation is not greater in the experts’ performance compared to the amateurs’ performance, regardless of whether the standard tempo is absolute or relative. This result of SD features for DBH Duration and DBH Articulation supports the evidence that a professional pianist is better at maintaining the balance of note durations and articulations between the hands. This outcome intuitively implies that professional pianists are better at temporally managing the coordination between their hands than amateurs [68, 73]. Keyboard instruments require the musician’s constrained hand and finger movements. The unique characteristics of keyboard instruments include the responsiveness, evenness, and resistance of the keys [52]. Pianists should be well aware of the attributes of these keyboard instruments and be able to independently control the movement of both hands and fingers. In the study of Lipke-Perry *et al*. [52], where participants performed a C-Major scale on three different pianos, professional pianists found that the pitch length pattern was consistent regardless of the difference in the key resistance of each instrument, the change direction of play, and the bimanual performance. This finding was influenced by differences in the motor control of the RH and LH rather than the comparison across piano instruments or the participants’ experiences (however, participants were pianists), and it suggests that to better understand piano technique, it is necessary to have a more detailed conception of neuromuscular function. Previous studies of the comparison of keystrokes by professional pianists and novice pianists have shown that the control and organization of complex multi-joint motor action are influenced by long-term extensive training from an early age [74, 78–83]. Professional pianists work to reduce muscle load and achieve the physiological efficiency of fatigue-prone muscle by using more proximal joints and gravity, inter-segment, and reaction forces than novice piano players [43, 84, 85]. In addition, professional pianists have a smaller increase in co-activation of hand and finger muscles than amateurs and less stiffness with tempo, and the use of elbow motions allow fingertip-key contact with low stiffness across a wide range of tempi [74, 86]. Thus, when playing the piano, the fact that the player can control the force of pressing the piano key with a small physical burden mean that the power of both hands can be controlled and is not greatly altered with the smaller co-activation of hand and finger muscles. Our findings show that only the Delta SD for two features differed significantly between experts and amateurs. Delta SD indicates the amplitude of change in the continuous playing feature between notes. In other words, this feature indicates how well the piano keys can be played continuously and smoothly in temporal and physical aspects. Specifically, the experts showed a significantly smaller SD than the amateurs related to the Delta of DBH in note duration. Therefore, this finding indicates that expert group are able to temporally manage hand-to-hand balance better than amateur group.

The DBH Attack Deviation indicates some notable aspects that reveal extreme differences between experts and amateurs. Specifically, the result for the DBH Attack Deviation mean demonstrates that experts tend to play their right-hand notes faster than their left-hand notes, while the amateurs tend to play their left-hand notes faster than their right-hand notes. These findings support previous research of *melody lead*, one of the phenomena that occur when both hands are performed asynchronously [33, 36, 37, 68]. Especially, this suggests consistent results as previous studies that professional pianists showed a larger melody lean (typically if the melody was on the right hand) than student pianists. Many university piano professors and skilled pianists believe that the LH is technically weaker than the RH, and the LH is slower than the RH [87–89]. This belief might also be supported by the fact that, dissimilar to previous Articulation interpretations, the unconscious reflection of the melody lead seems to cause performers to hit simultaneous notes asynchronously [33, 35, 36, 90]. However, the results of this study, it is not clear whether the performer seems that the most important voices (or the primary) were more emphasized by changing the timing of different voices (between the two hands) for intended expressive performance, or whether it is unconsciously performed of *melody lead*. The materials used in this study, the Hanon and the C-Major scale, are played in parallel with the RH and LH at an octave distance; that is, the octave is the only factor that differs between the two hands, and their melodic intonations are the same. Nonetheless, following the statement that expert piano playing can be described as a deliberate expression device [36, 68], the findings for the DBH Attack Deviation mean could confirm the conventional belief that professional pianists tend to show melody lead. This interesting result also suggests that the two proficiency groups differed in terms of the timing DBH, although all of the participants were right-handed.

### Dynamic attributes

Other notable results regarding the DBH in the two proficiency groups related to dynamics. First, the Velocity mean and SD features reveal a significant interaction effect between hand and group as well as significant main effects of hand and group. For the Velocity mean features, the expert group showed a greater DBH than in the amateur group, while the two proficiency groups showed similar tendencies in that the Velocity mean was greater for the right-hand notes than for the left-hand notes. It has been argued that the difference in velocity between the two hands occurs among professional pianists because the RH tends to play the melody, which is often given in the higher voice, and the LH tends to play the bass [33, 91]. Rubin-Rabson [92] noted that it is particularly easy to emphasize notes with the RH because the melodies are played mainly in the treble clef (G clef). In addition, we should note that the volume of the sound from a piano differs depending on the length and thickness of the corresponding string of the pressed key, even with the same magnitude of pressure [93]. Therefore, in order to give an outstanding piano performance, a pianist must be trained to deliberately control the magnitude of pressure to play the treble and bass keys and to express the melody and accompaniment parts differently. Especially for the two excerpts in this study, the RH and LH have a pitch difference with a wide range (one or two-octaves); the dynamic balance of the melody and accompaniment can differ depending on the control of the RH and LH. Hence, these excerpts might have requited more extreme control from amateurs, as the control of dynamic features between the RH and LH was more difficult to achieve for amateurs than experts. The volume is associated with a decrease in the anti-gravity activity of the elbow flexors and an increase in the activity of the elbow extensors in professional pianists and beginners [94, 95]. To produce a louder tone, professional pianists increased the amount of shoulder joint deceleration, leading to greater inter-segment dynamics, and beginners simply generated greater elbow muscle torque [81]. In other words, experts used “arm weights” to reduce elbow flexion or anti-gravity activities when pressing keys, while beginners used elbow muscles to achieve these goals [81]. In other words, when adjusting the loudness, the amateur uses different muscles than the professional to apply larger, longer forces and to press the piano keys while applying force to the fingers not directly touching the piano keys [83, 96, 97]. Thus, the pressing force is inefficient. Pianists’ long-term practice affects the motor tissue and muscle coordination in charge of volume control [43, 81, 94, 95]. Moreover, the practice time (i.e., deliberate practice) accumulated before the age of twenty by professional pianists has been reported to be an indicator of the performance of highly skilled movements [98]. After years of extensive piano education and intensive practice, experienced pianists are able to independently control both hands and fingers [99], dexterously control the strength of the hands, and detect the reaction of the piano to elicit a range of volume. The significance of the interaction effect in this study shows that the DBH was significantly different between the two proficiency groups. This conclusion can also be confirmed in the results of the DBH Velocity features showing that the expert group had a greater DBH than the amateur group. The result that the expert group has a greater dynamic DBH than the amateur group is because they are well aware of the need to handle both hands independently and differently, dexterously control the strength of the hands, and detect the reaction of the piano to elicit a variety of dynamic sound. This ability of a professional pianist is possible because it is ingrained in the body through years of intensive practice. As such, through years of professional education, the learning process and the amount of deliberate practice are factors that contribute to the ability of professional pianists to understand the various characteristics of the piano keyboard and to control the dynamics of melody and accompaniment.

**Fig 4:**
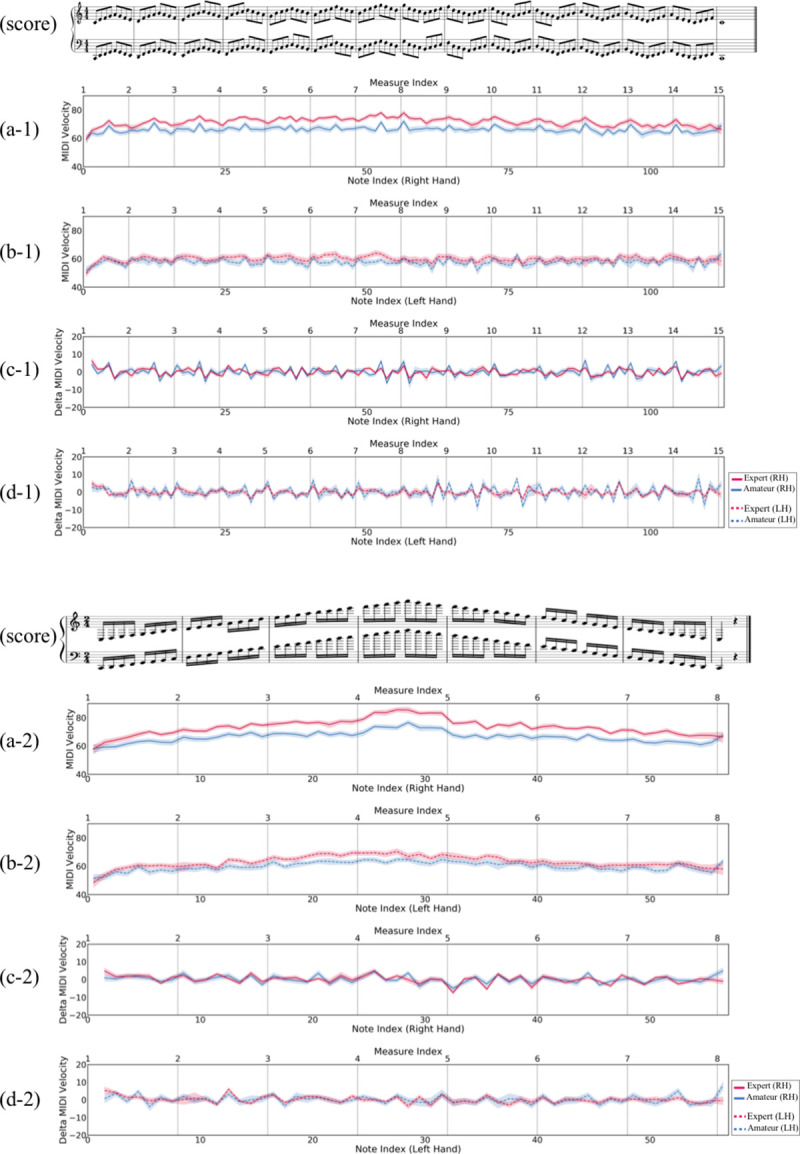
Two graphs that illustrate Velocity and its Delta of the performers between the two pieces. Each feature value is averaged among all of the performers in each proficiency group and is shown as a line in each graph. The shaded area around the line refers to the range of standard error for each note. The corresponding music scores are aligned with the graphs’ timeline to show how the feature values change relative to the musical patterns. In each graph, the red line indicates the expert group, and the blue line indicates the amateur group. The top musical score is "Hanon Exercises No. 1", and the bottom musical score is "C-Major scale". (a) and (c) show the right hand (RH)’s results with the solid lines, and (b) and (d) show the left hand (LH)’s results with the dotted lines. The boundaries of the measures are also depicted as vertical lines in the graphs.

For the Velocity SD features, DBH was also greater for the expert group than for the amateur group. However, it is interesting that the Velocity SD features of the amateurs showed the opposite direction from that of the experts in the slope between the corresponding mean values of the RH and LH. This outcome indicates that experts show a larger variance in the Velocity with the RH, while amateurs show a larger variance with the LH. Fig 4 provides some clues into this phenomenon by demonstrating the note-based changes in the Velocity mean that occurred when the performers played the two excerpts. According to this figure, at (a) and (b) in the Hanon and the C-Major scale, the experts changed the Velocity on the RH over a wider range than on the LH for both excerpts. Specifically, the RH of the experts started with a similar Velocity to that of the LH and reached a maximum Velocity that was much greater than of the LH. This type of hand control was stronger in the experts than in the amateurs. According to Wiley [89] and Parncutt *et al*.’s [69] contentions, when playing the piano in general, more attention is paid to the hand with the melody. In general, melodies tend to come from the upper voice, so experts have greatly altered the dynamics of the right hand to emphasize the melody and play the accompaniment with a relatively quiet sound. However, amateurs who have difficulty controlling both hands at the same time pay attention to the RH because they are interested in the hand playing the melody, and it is difficult to control the LH. The findings of this study support the belief of many professional pianists that piano students should focus on their LH [87–89]. At the same time, this result does not explicitly support the amateur group’s opposite trend from the expert group in the Velocity SD.

This trend in the amateurs can be proved by the Velocity Delta SD feature, which indicates how much the change in the Velocity is maintained over the performance. According to Fig 2, the Velocity Delta SD is much higher for the amateurs than for the experts. That is, the Velocity between successive notes tends to vary in a narrower range in the experts’ performance than in the amateurs’ performance. This outcome is also depicted in (c) and (d) of Fig 4. According to the figure, the experts are shown to change dynamics during the excerpt gradually so that the resultant Velocity follows the contour of the melody, regardless of the hand. In contrast, the amateurs show inconsistent dynamic changes in both hands that are less correlated with the melodic patterns. These results might have resulted in the experts having smaller Velocity Delta SD values than the amateurs. Meanwhile, the Velocity Delta SD features showed similar tendencies between the two proficiency groups because the corresponding mean value is higher for the LH than for the RH, with no significant interaction between hand and group. It can be assumed that the expert group’s deliberate control of the RH for the Velocity along the arch-like contour of the melody might have caused a large Velocity Delta SD in the LH to be compensated for by a larger Velocity SD in the RH, while the amateur group might not have had such compensation because the Velocity in the RH was not sufficiently correlated to the same melody.

The DBH Velocity features replicate the results from the Basic features. In particular, the DBH Velocity mean and SD are significantly higher for the experts than for the amateurs. Moreover, the DBH Velocity Delta SD is significantly different between the two proficiency groups. This result suggests that there is also a relationship with the melody lead mentioned in the results of the Articulation and DBH Attack Deviation features of Temporal attributes. A previous study on melody lead by Goebl and Repp explained that greater *melody lead* occurs when professional pianists play more largely dynamically separated melodies and accompaniments from asynchronous types [33, 68]. In conclusion, all of these findings demonstrate that the ability to control dynamics with both hands is a distinct aspect of professional pianists that can distinguish them from amateurs. This ability might also be indicative of a greater ability to express the given music dynamically. This outcome is also supported by the work of Bernays *et al*. [28], suggesting that there is a difference in dynamics (hammer velocity) between the hands—one of the various aspects that a professional player must master to effectively express the nuances of the timbre.

### Classification

Finally, we examined the effect of considering DBH in the classification tasks of determining performance proficiency. To this end, we defined the basic group with 48 Basic features and the DBH group with 16 DBH features as baselines and the Basic + DBH group with all 64 features as our experimental group. The classification results from the PCA and SVM for these groups showed that the mean *F* 1 scores are the highest when using both the Basic features and DBH features. These results are also supported by further investigation that removed the difference in the number of principal components among the feature sets. This outcome implies that explicitly considering DBH can efficiently add information to the classification model on how the two proficiency groups perform differently with the same given scores, even in cases in which the scores do not require any musical expressions. Thus, using the simple DBH features together with the standard performance features could improve quantitative performance evaluation systems that have previously addressed only conventional features, without the need to devise additional complex rule-based features.

## Conclusions

In this study, we explored how the balance between the two hands differs according to the proficiency level of piano players for quantitative performance evaluation. We extracted hand-crafted features from the performance MIDI data and rearranged them into two types of feature sets: Basic features, including RH and LH note-based features, along with their average (BH), and DBH features representing the direct difference between the LH and RH. We statistically analyzed these features according to the two proficiency groups, experts and amateurs, and examined the types of main effects or interactions of the features. These features were further used in a classification task to match each performance to the appropriate proficiency group to verify the importance of DBH for automatic evaluation of musical expertise. As a result, interaction effects in some of the temporal and dynamic properties suggested that the difference between the RH and LH observed in this study was more pronounced in the expert group than in the amateur group. In particular, the experts tended to press the piano keys longer and faster with the RH than with the LH, while the amateur group pressed the piano keys with a similar length and velocity regardless of the hand. Moreover, the experts tended to have greater DBH in dynamics than the amateurs. Furthermore, the group of experts showed regularity in MIDI velocity according to the melodic intonation in the score, whereas the amateur group was irregular. The results from the classification task also suggested that using the DBH features together with the Basic features could promote the accuracy of predicting proficiency groups compared to using only the Basic features. Since a number of conventional studies have claimed that controlling the balance of both hands is one of the critical skills for professional pianists, the results of this study demonstrate that the DBH differs depending on the proficiency level of the piano performance. We suggest that this approach could be an important tool for exploring quantitative piano performance evaluation, as well as piano performance analysis. Since it is a critical topic for automatic music assessment to find distinctive and representative properties to distinguish musical proficiency, this study is expected to guide effective solutions to this issue that can also be applied to piano education and further research into piano performance using machine learning.

## Supporting information

S1 Table Basic features and DBH features index. (PDF)

S1 Fig The results of ANOVA line graphs of Hanon and C-Major scale excerpts. rDuration and rIOI features.
(PDF)

S2 Fig The results of ANOVA line graphs of Hanon and C-Major scale excerpts. Articulation and Velocity features.
(PDF)
