# Supplementary material for: Quantitative analysis of piano performance proficiency focusing on difference between hands
Source: PLoS One. 2021 May 19;16(5):e0250299. doi: 10.1371/journal.pone.0250299 (PMC8133499; doi:10.1371/journal.pone.0250299)
Supplement: S1 Table — (PDF) [file pone.0250299.s001.pdf]

S1 Table. Feature indices for all 64 features

No.	Features	No.	Features
1	rDuration mean	37	Velocity mean
2	rDuration SD	38	Velocity SD
3	rDuration left hand mean	39	Velocity left hand mean
4	rDuration left hand SD	40	Velocity left hand SD
5	rDuration right hand mean	41	Velocity right hand mean
6	rDuration right hand SD	42	Velocity right hand SD
7	rDuration delta mean	43	Velocity delta mean
8	rDuration delta SD	44	Velocity delta SD
9	rDuration delta left hand mean	45	Velocity delta left hand mean
10	rDuration delta left hand SD	46	Velocity delta left hand SD
11	rDuration delta right hand mean	47	Velocity delta right hand mean
12	rDuration delta right hand SD	48	Velocity delta right hand SD
13	rIOI mean	49	DBH Duration mean
14	rIOI SD	50	DBH Duration SD
15	rIOI left hand mean	51	DBH Duration delta mean
16	rIOI left hand SD	52	DBH Duration delta SD
17	rIOI right hand mean	53	DBH Attack deviation mean
18	rIOI right hand SD	54	DBH Attack deviation SD
19	rIOI delta mean	55	DBH Attack deviation delta mean
20	rIOI delta SD	56	DBH Attack deviation delta SD
21	rIOI delta left hand mean	57	DBH Articulation mean
22	rIOI delta left hand SD	58	DBH Articulation SD
23	rIOI delta right hand mean	59	DBH Articulation delta mean
24	rIOI delta right hand SD	60	DBH Articulation delta SD
25	Articulation mean	61	DBH Velocity mean
26	Articulation SD	62	DBH Velocity SD
27	Articulation left hand mean	63	DBH Velocity delta mean
28	Articulation left hand SD	64	DBH Velocity delta SD
29	Articulation right hand mean		
30	Articulation right hand SD		
31	Articulation delta mean		
32	Articulation delta SD		
33	Articulation delta left hand mean		
34	Articulation delta left hand SD		
35	Articulation delta right hand mean		
36	Articulation delta right hand SD		
