# Supplementary material for: Quantitative analysis of piano performance proficiency focusing on difference between hands
Source: PLoS One. 2021 May 19;16(5):e0250299. doi: 10.1371/journal.pone.0250299 (PMC8133499; doi:10.1371/journal.pone.0250299)
Supplement: S2 Fig — Articulation and Velocity features. (PDF) [file pone.0250299.s003.pdf]

S2 Fig. ANOVA line graphs of comparisons between hands for basic features in expert and amateur groups.

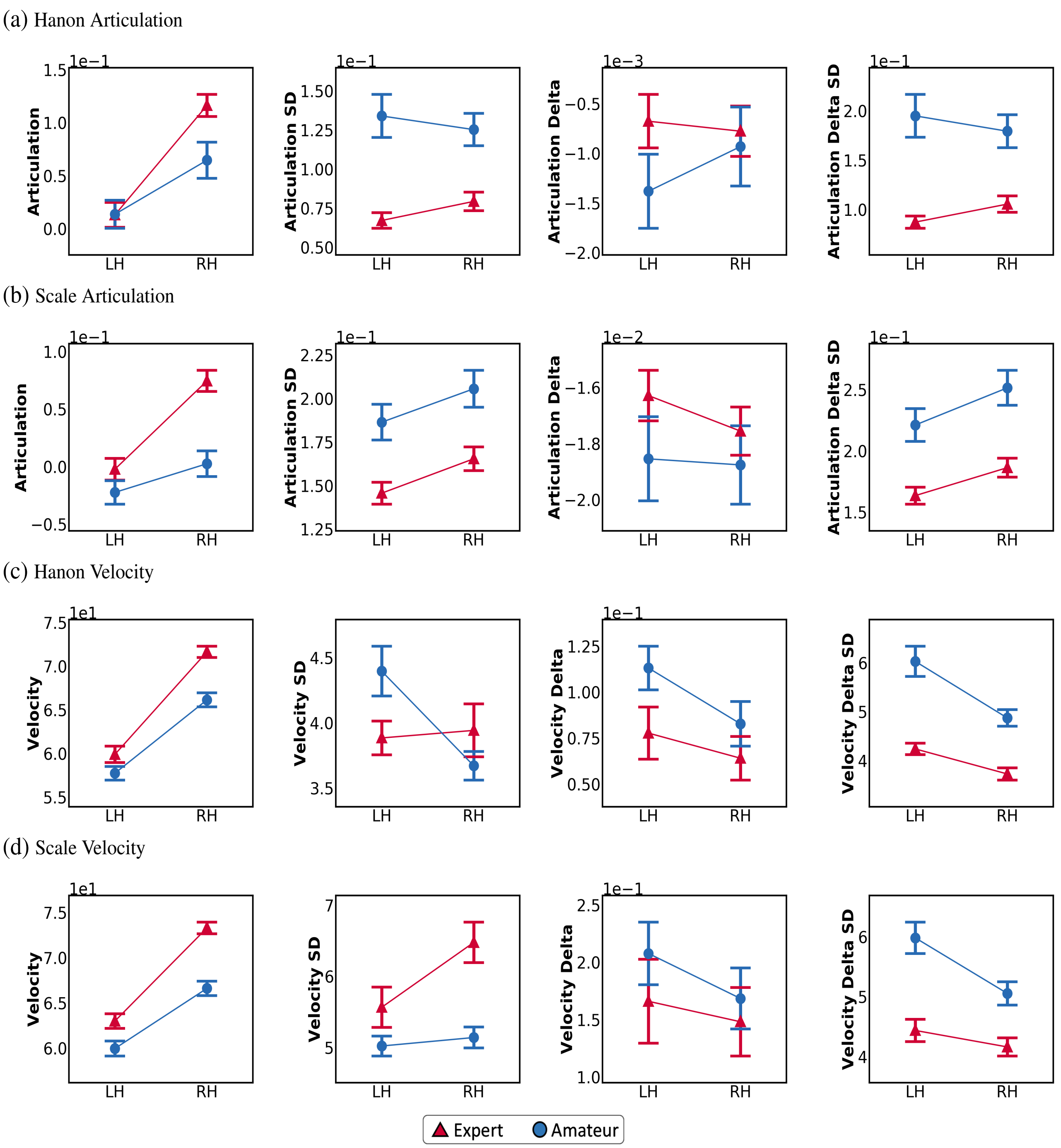

S3. These line graphs show the main effects for both groups. The graphs are results of the feature values from both excerpts ("Hanon" and "Scale"). (a) The Articulation features as mean, SD, Delta mean, and Delta SD of Hanon. (b) The Articulation features as mean, SD, Delta mean, and Delta SD of Scale. (c) The Velocity features as mean, SD, Delta mean, and Delta SD of Hanon. (d) The Velocity features as mean, SD, Delta mean, and Delta SD of Scale (expert, red; amateur, bule).
